# The promises and pitfalls of applying computational models to neurological and psychiatric disorders

**DOI:** 10.1093/brain/aww209

**Published:** 2016-08-20

**Authors:** Christoph Teufel, Paul C. Fletcher

**Affiliations:** ^1^ 1School of Psychology, Cardiff University, Tower Building, 70 Park Place, CF10 3AT, Cardiff, UK; aww209-2 2Cardiff University Brain Research Imaging Centre (CUBRIC), Cardiff University, Maindy Road, CF24 4HQ, Cardiff, UK; aww209-3 3Department of Psychiatry, University of Cambridge, Herchel Smith Building, Addenbrooke’s Hospital, CB2 0SZ, Cambridge, UK; aww209-4 4Cambridgeshire and Peterborough NHS Foundation Trust, Ida Darwin Site, Fulbourn, CB21 5EE, Cambridge, UK

**Keywords:** computational psychiatry, neuropsychiatry, schizophrenia, hallucination, delusion

## Abstract

Computational models have become an integral part of basic neuroscience and have facilitated some of the major advances in the field. More recently, such models have also been applied to the understanding of disruptions in brain function. In this review, using examples and a simple analogy, we discuss the potential for computational models to inform our understanding of brain function and dysfunction. We argue that they may provide, in unprecedented detail, an understanding of the neurobiological and mental basis of brain disorders and that such insights will be key to progress in diagnosis and treatment. However, there are also potential problems attending this approach. We highlight these and identify simple principles that should always govern the use of computational models in clinical neuroscience, noting especially the importance of a clear specification of a model’s purpose and of the mapping between mathematical concepts and reality.

## Teaser

Computational models are applied increasingly to the study of brain function and dysfunction. Teufel & Fletcher highlight the promise of this approach, but also some of the problems that can arise from the misapplication of such models. Using a simple analogy, they identify key principles necessary for avoiding common pitfalls.

## Graphical abstract

**Figure:**
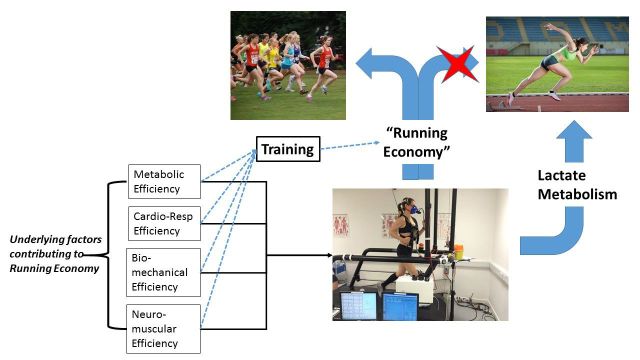
Computational models are applied increasingly to the study of brain function and dysfunction. Teufel & Fletcher highlight the promise of this approach, but also some of the problems that can arise from the misapplication of such models. Using a simple analogy, they identify key principles necessary for avoiding common pitfalls.

## Introduction

Though frequently implicit, models are ubiquitous in science. If successful, they allow us to make complex problems more tractable by simplifying them to a set of deep, hidden components that are the main drivers of the visible phenomena the model attempts to explain. This leads to a seemingly paradoxical situation: a model must necessarily neglect many aspects of reality to represent adequately the deeper causal structure of that reality. Thus, even at the earliest stages of modelling, we are forced to make two important assumptions: first, that certain aspects of reality can be ignored because they are irrelevant to the bit of reality that the model attempts to explain; second, that there are parts of the model that ‘stand for’ things in a way that is meaningful and useful (despite the fact that the model is necessarily an incomplete rendition of reality). Ultimately, therefore, the value of modelling depends upon a clear conceptualization of, and adherence to, the mappings between the model and reality and this, in turn demands a careful consideration of when and where it applies. Incorrect models can be heuristically useful but incorrect application of models will be misleading.

In the modern brain sciences, the most powerful models are computational in nature. Researchers in computational neuroscience draw on a wide range of disciplines and tools with the aim of constructing formal mathematical models of neurobiological and mental processes ( Dayan and Abbott, 2001 ). The relatively novel approach harnesses these powerful computational methods and applies them to psychiatric and neurological disorders ( Maia and Frank, 2011 ; Montague *et al.* , 2012 ; Corlett and Fletcher, 2014 ; Friston *et al.* , 2014 ; Stephan and Mathys, 2014 ). It thus lies at the interface between computational and cognitive neuroscience, psychology, and psychiatry/neurology. But computational clinical neuroscience is by no means a homogenous field: its models differ in their intended purpose, the mathematical techniques employed, and the level of explanation they seek, ranging from mechanistic or process models of neural circuits to abstract normative models of high-level mental function. Despite this heterogeneity, however, the common hope and promise is that these models will provide a deeper understanding of the neurobiological and mental processes that contribute to psychiatric and neurological disorders and, ultimately, be key to progress in diagnosis and treatment.

While we share the growing enthusiasm for explicitly modelling the processes of the mind, and their disruptions, in mathematical and computational terms, we cannot help but notice an evolving feeling of disorientation and puzzlement in some observers of the field. In particular, there seems to be a growing sense that computational psychiatry in particular, while developing in several different directions at once, is making assertions that are often highly mutable, opaque, and, paradoxically, given the intentions, inexact. Here, therefore, we scrutinize afresh the general nature of modelling and ask ourselves how we may determine whether our models are serving us or misleading us. We begin by outlining what we consider to be the three most important benefits of computational models in psychiatry, neurology and, indeed, clinical neuroscience generally: (i) enforcing rigour and precision in the formalization of conceptual models; (ii) inspiring useful new conceptualizations of known phenomena and providing a principled means of synthesizing disparate pieces of evidence by helping to identify core principles of brain disorders; and (iii) offering a means of bridging the gap between different levels of explanation all the way from basic neurobiology to conscious experience of suffering. Such powerful insights can, if generalized, offer profound new ways to make predictions about the brain but the potential benefits come with serious challenges, which we will highlight. We will identify key principles and criteria, which, though well known in the field of modelling, are easily neglected with appreciable cost. By applying these principles scrupulously, we argue, the researcher can harness the power of the modelling approach while avoiding the dangers of drawing unwarranted inferences.

## The value of computational models in understanding brain function and dysfunction

**Figure 1:**
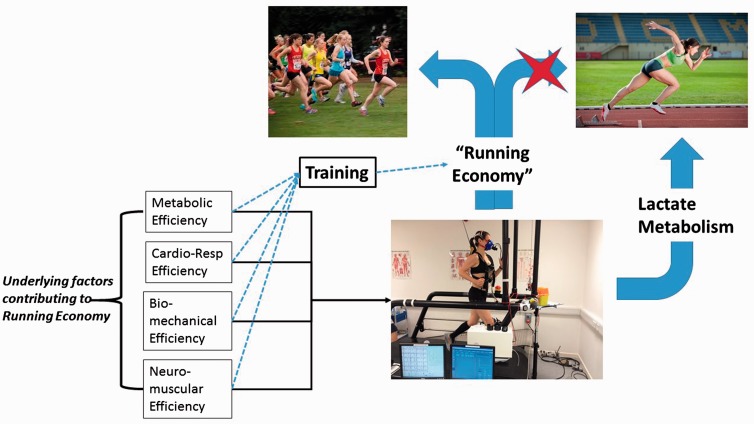
**The importance of specifying both the purpose of a model, and the mapping between the model components and aspects of the ‘real world’.** Here, we illustrate the importance of two principles of modelling using the running analogy from Box 1 . It is tempting, when we have identified a reliable measure, one that seems to capture the essence of what we are trying to characterize and that has predictive value, to make it generally applicable. We may forget or ignore both the elements of the world that we are not including in our model and, furthermore, the ways in which the components that we are modelling may map to the real world. The figure illustrates how we might use ‘Running Economy’ (the volume of oxygen consumed at steady state running) as described in Box 1 as a predictor of race performance. Having the figures for oxygen consumption on two athletes allows a direct comparison of how they are likely to fare in competition. Indeed, even if the measurements were obtained at a range of speeds and on different terrain, the measures may be comparable. Running economy serves as a good model of running ability. But, as indicated in the figure, it only fulfils this purpose within predefined constraints. For example, it is standard to measure running economy at a pace that is below that at which a person is near their lactate threshold (the point at which oxygen consumption cannot keep up with demand) and therefore the model is inadequate if we wish to judge running ability for shorter distance sprints in which lactate levels are highly relevant ( Billat, 1996 ). Here the mapping between the model and reality has gone wrong because the model is applied for a setting where its assumptions are no longer relevant or valid. Unless we are explicit about our mapping between model components and reality and the working assumptions that justify it, our model will ultimately lead us astray. Such error lies not in the model but in how it is used. It should also be noted that running economy, even when applied under restricted and appropriate circumstances, emerges from a number of complex, interacting factors that are individually ignored, though each contributes to the overall measure (indicated by straight black arrows). This is not a problem when running economy is being used for the purpose of predicting running performance but it may become very important if we are using our model for a different purpose, for example to decide on the best training regimen to improve performance. At this point, the value of the model is much more influenced by the factors that were previously only an implicit part of the model. Two athletes may have limitations in their running economy for very different reasons, one due to biomechanical inefficiency arising from flaws in her posture or stride rate, another due to cardiovascular inefficiency. Each would be rectified by distinct training regimens (as indicated by blue, dotted arrows), which could not be chosen merely on the basis of running economy. It is the purpose of the model that specifies which components of reality need to be modelled and in how much detail.

Below, we discuss three consequences of using computational models that we believe are most relevant for clinical neuroscience, and illustrate these with a simple analogy in Box 1 and Fig. 1 .

Box 1 Benefits of computational modelling: a simple physical analogy

This example aims to illustrate what we might aspire to in developing our models of brain function with a simple analogy. In addition, it helps to highlight the core principles of computational modelling and the perils of departing from them ( Fig. 1 ).

Suppose that you are interested in training a runner. You want to know how good she currently is, how she compares to other athletes, whether she is performing at her optimal level or could benefit from a training regimen and, if so, how this might best be structured. You might begin with a very simple—but workable—conceptualization of her ‘fitness’, perhaps defined as how far she might run at a given speed and you might note that the more your athlete runs, the fitter she gets. But, after a time, she reaches a point where there is no further improvement. Has she reached her limit? How might a more formal, mathematically-informed approach help you to determine this and to find ways of producing further improvements?

**(i) Developing rigor and precision**

In setting up a more complex model of what makes good runners, you would gain important benefits. It would encourage you to identify more precisely the key components (at multiple levels) of the act of running. You may come to see the relevance of the biomechanics of her running—such as stride length and cadence—as well as muscle and fat distribution, cardiovascular markers and subjective factors such as pain and motivation. More detailed measurements could provide markers for underlying metabolic processes—her maximal oxygen consumption and her lactate threshold as well as microanatomical factors such as proportions of slow- and fast-twitch muscle fibres. Importantly, through mathematically modelling these variables and, in particular, how they relate to each other, you have gained a much deeper understanding that might allow you to identify important, but previously unconsidered, factors driving performance. The model provides too a more powerful tool to assess the impact of different training regimens.

**(ii) A new conceptualization**

The original simple conceptualization of fitness (maximum distance covered at a given pace) captures some aspects of the athlete’s ability but it only indirectly captures her race performance. In drawing together the biomechanical, biochemical and physiological levels in a mathematical model you find that a more useful conceptualization emerges, one that has much greater capacity to distinguish between runners. For example, her ‘running economy’ ( Barnes and Kilding, 2015 ) (amount of oxygen consumed per kg per min), may be more suitable for your purposes, offering both a predictive measure and one that you can focus on in improving her training.

**(iii) Bridging the gaps**

This new conceptualization, and the modelling process that generated it, provides a link across multiple levels of description of the act of running: the biomechanical, the neuromuscular, the biochemical and the physiological. All factors may play a part, both separately and through their interaction, in determining oxygen consumption per kg per min. Exploring its mathematically-defined relationships to changes at these many levels may offer new and powerful ways to understand, assess, and, ultimately, change running performance.

### Enforcing precision through formalization

The formalization of existing conceptual models in mathematical terms is a way in which computational models contribute to our understanding of processes in the mind and brain. Formal models provide several important advantages over purely conceptual ones. First, conceptual models that are phrased exclusively in linguistic terms inevitably carry a certain amount of vagueness and ambiguity, independently of how advanced and detailed the technical terminology used to express them. In the ideal situation, the act of translating conceptual ideas into mathematical terms enforces a rigorous and precise way of thinking that adds specification to the conceptual model. It is particularly useful in forcing the researcher to explicitly specify which components are relevant (and which are irrelevant) and how these components are related. Often this has the additional benefit of helping to uncover implicit assumptions that might remain unnoticed without such formalizations.

To illustrate, consider the experimentally measured tendency of individuals with schizophrenia to sample less evidence than healthy controls before reaching a decision when confronted with a probabilistic, uncertain environment. Since its initial discovery ( Huq *et al.* , 1988 ), this jumping to conclusions (JTC) bias has received a large amount of attention ( Fear and Healy, 1997 ; Garety *et al.* , 2005 ; Corcoran *et al.* , 2008 ) as an interesting and informative phenomenon in developing our understanding of delusional thinking. A widely accepted hypothesis assumes that the JTC bias reflects an expectation of high costs for sampling new pieces of information. However, a recent mathematical formalization that explicitly modelled sampling costs as one of the variables underlying decision-making suggests that this view, which implicates a difference in subjective motivational factors, is not supported by a more carefully formalized model ( Moutoussis *et al.* , 2011 ). Rather, the model indicates that noise in decision-making is a key factor in the JTC bias. While the nature of this noise and its precise alteration in delusions, is not yet clear, it may be that it reflects neural noise, known to be a key factor in neural information processing and previously ignored in the JTC. The key point here is that its importance in explaining the JTC bias had been easily overlooked until a quest for an explicit formalisation of the bias ruled out an alternative view and demanded its consideration.

Another illustration of the usefulness of computational models in shaping our understanding of brain disorders comes from neurological work that evaluates the consequences of lesions to brain areas involved in high-level vision ( Plaut and Behrmann, 2011 ; Behrmann and Plaut, 2012 ). Addressing the debate about whether visual recognition is carried out in dedicated, category-specific modules or general-purpose mechanisms, Plaut and Behrmann (2011) constructed an artificial neuronal network of face and word recognition. Rather than supporting either interpretation—dedicated, category-specific modules versus general purpose mechanisms—the simulation combines these views and shows how a graded specialization of computer-simulated brain areas emerges naturally as a consequence of general computational principles. Without the computational model, it would have been most likely more difficult to clearly specify such a middle-ground solution to the debate. Importantly, based on this model, the authors predicted a specific pattern of face and word processing impairments in prosopagnosia and pure alexia, which was subsequently confirmed empirically ( Behrmann and Plaut, 2012 ).

These examples illustrate the crucial heuristic value of computational models and their use as a tool for structuring our thoughts and explanations. It demonstrates how readily computational formulations offer and encourage an ever more detailed and quantitatively precise characterization of neurobiological, behavioural, and mental processes, highlighting the potential importance of previously ignored parameters, suggesting new experiments, and more sophisticated explanatory frameworks. Such an iterative process is likely to be crucial as neurology, neuropsychology, and psychiatry strive towards a more mechanistic understanding of symptoms. In psychiatry, this is particularly important in helping to critically review its diagnostic categorizations from the perspective of a dimensional approach to illnesses of the mind and brain ( Montague *et al.* , 2012 ) (also see Box 2 ). The fact that existing diagnostic categories group biologically heterogeneous syndromes, with potentially different pathophysiological mechanisms, into one disorder constitutes a real obstacle to developing an understanding of the neurobiological underpinnings of mental illness ( Cuthbert and Insel, 2013 ). Explanations based on computational models seek to go deeper than current conceptualizations, with the aim of describing these symptoms at lower levels, and thereby uncovering potential mechanisms underlying symptom clusters independent of diagnostic category. Thus, computational models may be a necessary prelude to an improved classification system in psychiatry ( Box 2 ).

Box 2 Computational modelling giving new perspectives on diagnosis and treatment

One of the most profound challenges facing psychiatry is its reliance on a diagnostic system that is both widely distrusted and criticized and, at the same time, forms the basis for most research and clinical practice ( Cuthbert and Insel, 2013 ). The central problem is that diagnostic categorizations are based on phenomenological similarity, often expressed at the level of perception, belief and emotion. Consequently, there is a danger that superficially similar, but fundamentally different dysfunctions may be grouped together under one category while some categorical distinctions might be based upon superficial differences that do not actually reflect underlying differences in pathology. The conundrum is reflected in the fact that many researchers and clinicians now challenge the idea that schizophrenia meaningfully refers to a particular group of people ( van Os, 2016 ) while, conversely, the long-treasured distinction between schizophrenia and affective disorder is increasingly questioned ( Kendell and Jablensky, 2003 ).

Only through more precise characterizations of mental and brain function can we begin to converge on mechanisms that may allow us to distinguish between conditions that, despite superficial similarity, are actually fundamentally different. And it is only through identification of such mechanisms that we can avoid the creation of false distinctions between conditions that, though superficially distinct, are actually overlapping. The three benefits of the computational approach identified in the text and exemplified in Box 1 therefore become powerful allies in the pursuit of a credible diagnostic system. To return to the simple running analogy in Box 1 , it is easy to see how two runners may show similar levels of fitness or unfitness for different reasons, and how differences in current fitness might not reflect differences in core abilities. Only through more precise modelling of the underlying factors is it possible to understand how superficial resemblance may disguise lower level distinctions.

### Shaping novel conceptualizations and syntheses

Concepts derived from computational neuroscience have often led to exciting new ways of thinking about established phenomena in neuroscience more generally. For instance, classic studies in visual neuroscience have shown how notions derived from information theory ( Shannon, 1948 ; McKay, 2003 ) can provide compelling functional explanations of the specific structure of receptive fields in many peripheral visual systems ( Srinivasan *et al.* , 1982 ) or the shape of transfer functions in early visual neurons ( Laughlin, 1981 ). Moreover, the application of computational methods has often helped to synthesize disparate phenomena within a common explanatory framework. One remarkably successful and classic example of such a synthesis comes from Rao and Ballard (1999) , who demonstrated that a simulation based on a predictive coding scheme inspired by information theory reproduced a number of known receptive field effects. As well as showing the power of feedback modulations in the encoding of natural images, this model has been enormously influential in shaping ensuing experiments—and their interpretations—in visual neuroscience and beyond. It gives a glimpse of the unifying potential of computational modelling, a potential that has played a part in inspiring a remarkable growth in the use of such models in our attempts to understand brain dysfunction.

In psychiatry, Fletcher and Frith (2009) recently provided an integrated account of positive symptoms in schizophrenia—hallucinations and delusions—based on ideas derived from computational neuroscience. Conceptual models of schizophrenia have largely treated perception and belief as arising from distinct processes; it has consequently been hypothesized that hallucinations and delusions are caused by abnormal perceptual processing ( Maher, 1974 ), abnormal belief formation ( Huq *et al.* , 1988 ), or both ( Coltheart, 2007 ). Based on concepts originating in Bayesian decision theory ( Chernoff and Moses, 1959 ; Berger, 1985 ; Young and Smith, 2005 ), and earlier work implicating prediction error in the emergence of delusional beliefs ( Corlett *et al.* , 2007 ), Fletcher and Frith (2009) suggested a synthesis of these explanations, arguing that the unusual perceptual experiences and beliefs in psychosis can be explained by one core atypicality, namely a shift in the balance of Bayesian inference within a hierarchically-organized information processing system. By identifying a core principle, the model offers a parsimonious explanation for the co-occurrence of hallucinations and delusions as well as the fact that hallucinations and delusions are often not clearly distinct from each other. Moreover, it provides a common framework for understanding the emergence of positive symptoms from various different perspectives, linking levels of explanation that range from basic neurobiology to the individual phenomenology of suffering (see next section).

In this case, ideas derived from computational neuroscience—a multi-level, hierarchical predictive processing system—militate against a simplistic distinction between perceptual experience and belief and, by extension, the dichotomization of positive symptoms into hallucinations and delusions. Moreover, the model offers encouragement to move away from static characterizations of symptoms, suggesting rather that we must consider a dynamic balance in which prior knowledge is used to make sense of incoming sensory information, which either accords with or challenges that prior knowledge. This informational conversation occurs over time and at multiple levels with the overall system acting in pursuit of optimal predictions. The same core disturbance can thus give rise to different symptoms depending on the hierarchical level, at which it expresses itself. For instance, Teufel and colleagues (2015) have recently shown that, in both early psychosis and psychosis-proneness, there is a tendency for visual processing of ambiguous stimuli to show increased reliance on prior knowledge. While this finding is specific to atypical perceptual experiences rather than psychosis in general, the authors point out that such an effect, measured at one level of processing and one point in time, is likely to propagate up and down the hierarchy, inducing changes to inference systems at other levels in the brain. This growing and increasingly influential way of thinking about psychosis ( Aleman *et al.* , 2003 ; Corlett *et al.* , 2009 ; Fletcher and Frith, 2009 ; Chambon *et al.* , 2011 ; Adams *et al.* , 2013 ; Schmack *et al.* , 2013 ; Jardri and Denève, 2014 ; Teufel *et al.* , 2015 ; Fineberg and Corlett, 2016 ) is partly inspired by new ideas emerging during the search for overarching neurocomputational models.

### Bridging the explanatory gap

A widely held but contentious notion is that computational techniques might allow researchers to link various levels of explanation of mental illness ranging from basic neurobiological systems to the phenomenology of specific symptoms ( Huys *et al.* , 2011 ; Maia and Frank, 2011 ; Montague *et al.* , 2012 ; Friston *et al.* , 2014 ). While different levels of explanation may be independent, as most famously highlighted by David Marr (1982) , the hope is that computational models nevertheless will help to constrain and link descriptions at various levels. This linking of multiple levels of explanations will be critical in assessing different risk factors and their interaction in psychiatric illness ( Kendler, 2012 ), and, while advances so far in this regard are still speculative and incomplete, the potential for real progress is there.

As an example, there is a long tradition linking features of schizophrenia to basic associative learning. In this form of learning, knowledge acquisition is thought to be driven by so-called prediction errors, i.e. the difference between expected and actually experienced rewards. Of equal importance, schizophrenic symptoms have been linked to dopaminergic dysfunction ( Kapur, 2003 ). The formalization of associative learning in terms of computational models derived from machine learning ( Sutton and Barto, 1998 ) has been instrumental in relating these two levels of explanation ( Montague *et al.* , 1996 ). In particular, they provided the tool to link the formation of delusions to atypicalities in phasic dopamine via the prediction error signal that drives learning. For instance, computational models enabled researchers to relate psychotic symptoms in an animal model to dopaminergic neurons down to the receptor level ( Smith *et al.* , 2007 ) and, using functional MRI in psychotic patients, to abnormal activity in dopaminergic neurons in mesolimbic brain structures ( Murray *et al.* , 2008 ). Moreover, a series of complementary studies (for reviews see Frank, 2008 ; Maia and Frank, 2011 ), drawing on the advantages of different approaches (cognitive tasks, exploration of patients with known dopamine dysfunction, functional perturbations using deep brain stimulation and pharmacological manipulations) have demonstrated the links between dopaminergic neuronal populations, regional brain responses, precise cognitive functions and neuropsychiatric symptoms. Critically, these studies explored links suggested by, and hypotheses based on, computational reinforcement-learning models.

Another powerful example of how the key attributes of computational modelling may be harnessed to provide both a new conceptualization and an opportunity for multi-level explanatory insights, ultimately applicable to understanding neurological dysfunction, is seen in the work of Ueno and colleagues (2011) . Synthesizing a series of existing computational models, they applied known neuroanatomical constraints to generate a mapping between computational processing and brain anatomy. The ensuing ‘fusion of neuroanatomy and computation’ provided a new conceptualization with remarkable capacity to shed light on both normal language function and aphasia.

## Challenges

Though we recognize the huge potential offered by applying computational models to the study of mental and neurological illness and we share the growing enthusiasm for the field, there is a danger that these models obscure rather than clarify, and that they may form the basis for assertions that are frustratingly mutable and inexact. Consider for example, the application of Bayesian models to understanding hallucinations. Bayesian models are one way of formalizing the notion that our perceptual experience is the result of a combination of sensory evidence with prior knowledge and expectations of our environment. There is a growing body of empirical research that supports the idea that this framework is a useful tool to understand hallucinations. Yet, the general notion underlying Bayesian models has been invoked to support several profoundly different conceptualizations of how hallucinations may arise. First, an increased weighting of prior expectation in perception, such that expectations generate inaccurate percepts ( Friston, 2005 *b* ; Corlett *et al.* , 2009 ; Fletcher and Frith, 2009 ; Frith and Friston, 2013 ; Teufel *et al.* , 2015 ), second, a reduction in the relative weighting of prior expectation such that it is the relatively stronger bottom-up signal that generates the aberrant percept ( Adams *et al.* , 2013 ), and finally, a circularity in inferential processing such that a lack of inhibitory control generates a reverberating effect: an expectation enhances a signal which then acts as additional evidence in favour of that expectation ( Jardri and Denève, 2013 , 2014 ). While some of these models might not be mutually exclusive, clearly, a computational framework that is able to encompass such differing possibilities needs close scrutiny.

A related illustration of the difficulties facing the computational approach to neuropsychiatric illness comes from work attempting to relate high-level Bayesian models of brain function to processes at the neuronal level. Under certain specific but plausible assumptions, Bayesian models find a natural implementation in predictive coding schemes. In its original form, predictive coding provides a functional theory of perceptual processing located at an intermediate level between low-level mechanistic models and high-level explanations ( Spratling, 2008 *a* , *b* , 2013 ; Huang and Rao, 2011 ). More recently, some authors have embarked on a further step, treating predictive coding schemes as mechanistic models of information processing in the brain more generally ( Friston, 2005 *a* ). This perspective has been adopted in some models of psychotic experiences ( Adams *et al.* , 2013 ), perhaps offering the exciting possibility of making assertions about their precise neural instantiation. Here, based on a specific conceptualization of predictive coding, psychotic symptoms are thought to be a consequence of abnormal neuromodulation of post-synaptic gain of superficial pyramidal cells. This is an admirably precise and specific assertion and it depends on a correspondingly precise conceptualization of predictive coding. However, given that predictive coding is a functional scheme, other conceptualizations are possible ( Spratling, 2013 ; Kogo and Trengove, 2015 ). Indeed, it has been demonstrated that an alternative predictive coding model that, in some respects, is based on the opposite assignment between mathematical concepts and neurobiological implementation is not only mathematically equivalent to the model mentioned above but is well-supported by empirical data ( Spratling, 2008 *a* , *b* , 2010 ).

The above example simultaneously illustrates the promise of the computational approach to clinical neuroscience and the challenges inherent in the move towards drawing together a theory (Marr’s computational level) and a specific implementation of that theory (Marr’s physical level) ( Marr, 1982 ). The promise lies in the opportunity to explore computational ideas in terms of their neural mechanisms and the challenge is to avoid overburdening a model with interpretations that it cannot unambiguously support. Computational neuroscience offers us the tools and the framework to identify and resolve the discrepancies and ambiguity that the approach itself throws up. However, in order for the field to advance, it will be critical not to stretch the explanatory power of models beyond a certain point. This brings us back to the importance of being clear on what the purpose of our specific models is, which components of reality we are choosing (and neglecting) in building them, and in what sense the components of the model represent reality.

## A principled approach

In its ideal form computational modelling provides novel and powerful tools to shed light on information-processing atypicalities in neurological and mental illness. However, the approach also comes with difficulties and ambiguities. We suggest that many pitfalls could be avoided through use of two relatively simple and well-established, but often neglected, principles of modelling ( Bender, 1978 ). The first is that it is vital to specify clearly the purpose of a model and its role in the explanatory process ( Fig. 1 ). For instance, normative optimality models that can act as a benchmark against which to compare human performance, of course, serve a different purpose from mechanistic models of neurobiological or mental processes, or simulations that are supposed to provide a proof of principle ( Maloney and Mamassian, 2009 ). An important aspect of the process of clarifying a model’s purpose is a specification of the level of explanation or description at which the model represents reality. Second, and very closely related to the first point, is the importance of an explicit treatment of the mapping between mental or neurobiological process and mathematical concept ( Fig. 1 ). It is important to note ( Box 3 ) that we distinguish between a model being wrong in useful and in useless, or misleading, ways. A model need not be right (or indeed complicated) to be useful nor should it be constrained to such a degree that it cannot be used to generalize and predict beyond the confines of its initial application. However, its use must always align with the question or purpose that it is used for, and its relevance must be continuously evaluated. Only if it is very clear which mathematical concept ‘stands for’ which aspect of reality, can the validity of the model truly be assessed. In particular, the mathematical concepts need to be carefully chosen in such a way that they capture those aspects of reality that are essential to the chosen purpose of the model. Ultimately, the usefulness of a computational model is limited by how meaningful the relationship is between linguistically expressed concepts of reality and mathematical terms.

Box 3 Simple, general, and breakable: three useful properties of a good computational model

Good computational models are often simple. To be useful in the generation of explanations, models have to reach beyond the confusing diversity of reality and identify key causal components. Indeed, the process of simplification is itself useful. The act of choosing which parts of reality to include and which to exclude demands that the modeller is explicit about the model’s assumptions and limitations. Though computational modelling should strive for specificity and precision, detailed and isolated explanations of specific phenomena may ultimately impose a serious limit on the value of the model. Rather, one of the main strengths of computational models is their usefulness in uncovering deep organizational principles of a system, achieved by balancing simplicity with situation-specific accuracy.

The process of simplification thus embodies a quest for more universal principles that extend beyond the current situation. It thereby furthers the development of an explanatory framework that generalizes across, and provides prediction of, a whole range of phenomena. Such generalization is of course a hallmark of a successful model. It is important to keep in mind, however, that the process can easily become misleading when a successful model is applied to new domains without a detailed evaluation of its applicability. Thus, in addition to seeking the balance between simplicity and detail, we advocate a specific and precise treatment of the relationship between the model’s components and to-be-modelled aspects of reality, and of the model’s purpose in the explanatory process.

A focus on specificity and precision also does not mean that models should be abandoned if they are unsuccessful. Rather, failures should provide the impetus for an iterative process of updating, leading to novel experiments and further model development. It may be profoundly useful and informative for a model to break. Note that only a model that can make specific, testable prediction will be breakable. As with conceptual models, inadequately specified computational models may bend themselves, appearing to fit a wide range of data and, in doing so, become devoid of the attributes that ultimately make models useful.

## Concluding remarks

The use of computational models to understand brain function and its disruption is an exciting development. It promises to provide insights, in unprecedented detail, to psychological and neurobiological subsystems that go awry in disorders of the mind and brain, laying the foundation for the development of novel diagnostic systems and treatments that accurately reflect underlying causes. But the approach carries too the power to mislead and a mature, and ultimately successful use of computational models requires a detailed appreciation of their strengths and limitations as well as the simple principles that govern this use. Foremost, we must repeatedly ask ourselves, what are we modelling, how do our model components relate to reality and, crucially, what are we leaving out?

## Funding

This work was funded by the Wellcome Trust and the Bernard Wolfe Health Neuroscience Fund.

## Abbreviation

JTC: jumping to conclusions
